# Quantifying muscle glycosaminoglycan levels in patients with post-stroke muscle stiffness using T_1ρ_ MRI

**DOI:** 10.1038/s41598-019-50715-x

**Published:** 2019-10-10

**Authors:** Rajiv G. Menon, Preeti Raghavan, Ravinder R. Regatte

**Affiliations:** 1 Bernard and Irene Schwartz Center for Biomedical Imaging, New York University School of Medicine, New York, NY USA; 2 Present Address: Depts. of Physical Medicine and Rehabilitation and Neurology, Johns Hopkins University School of Medicine, Baltimore, MD USA; 3 Rusk Rehabilitation, New York University School of Medicine, New York, NY USA

**Keywords:** Skeletal muscle, Biomarkers

## Abstract

The purpose of this study was to provide imaging evidence of increased glycosaminoglycan (GAG) content in patients with post-stroke muscle stiffness; and to determine the effect of hyaluronidase treatment on intramuscular GAG content. In this prospective study, we used 3D-T_1ρ_ (T1rho) magnetic resonance (MR) mapping of the upper arm muscles to quantify GAG content in patients with post-stroke muscle stiffness before and after hyaluronidase injection treatment. For this study, healthy controls (n = 5), and patients with post-stroke muscle stiffness (n = 5) were recruited (March 2017–April 2018). T_1ρ_ MR imaging and Dixon water-fat MR imaging of the affected upper arms were performed before and after off-label treatment with hyaluronidase injections. T_1ρ_ mapping was done using a three-parameter non-linear mono-exponential fit. Wilcoxon Mann-Whitney test was used to compare patients’ vs controls and pre- vs post-treatment conditions. The T_1ρ_ values in the biceps were significantly higher in patients before treatment (34.04 ± 4.39 ms) compared with controls (26.70 ± 0.54 ms; P = 0.006). Significant improvement was seen in the biceps of patients before (35.48 ± 3.38 ms) and after treatment (29.45 ± 1.23 ms; P = 0.077). Dixon water-fat distribution was not significantly different in the patients compared to the controls (biceps P = 0.063; triceps P = 0.190). These results suggest that T_1ρ_ mapping can be used to quantify GAG content in the muscles of patients with post-stroke muscle stiffness, and that muscle hyaluronan content is increased in stiff muscles compared with controls, providing imaging corroboration for the hyaluronan hypothesis of muscle stiffness.

## Introduction

Stroke is a leading cause of disability in adults, with a total direct and indirect economic cost of over $40 billion in the US in 2013–2014^1^. Spasticity and muscle stiffness are common sequelae following stroke, with reported prevalence of 19–92%^2–4^. Post-stroke spasticity and muscle stiffness severely affect the quality of life of individuals with stroke and their caregivers, and drive up direct healthcare costs four-fold during the first year alone^5^. In addition, muscle spasticity and stiffness are also problematic for patients with multiple sclerosis, traumatic brain injury, spinal cord injury and cerebral palsy^6–9^.

The generally accepted neural mechanism underlying spasticity is a decrease in suppression of the spinal stretch reflexes resulting from reduced cortical influence on the spinal cord, causing hyperreflexia and muscle over activity^10^. Following neural injury, patients with spasticity and muscle stiffness also have weak muscles^11^ and conventional treatment of muscle over activity can further increase muscle weakness^12^. Furthermore, while hyperreflexia develops relatively early after neurologic injury, muscle stiffness develops over the ensuing weeks and months^13^. This increases the resistance to movement making it more challenging to move and participate in therapy. Non-neural mechanisms have therefore been proposed to explain the increased resistance to movement which may result from secondary changes in the structure or function of skeletal muscles secondary to the neural injury^14^. However, it is not clear how muscle properties change as a result of the neural injury^15^. The hyaluronan hypothesis proposes that the accumulation of the hyaluronan, within the extra-cellular matrix (ECM) of muscles contributes to the development of muscle stiffness^16^. Hyaluronan is a high molecular weight GAG in the ECM, which acts as a lubricant to aid the sliding of muscle fibers and bundles during movement^17^. Histological studies using animal models have shown that immobilization of skeletal muscle leads to the accumulation of hyaluronan^18^. Following a stroke, atrophy of muscle fibers leads to an increase in the ECM relative to the muscle fibers^19^, perhaps due to the accumulation of fat or hyaluronan, but this has not been shown conclusively. However, a recent case series showed that off-label, intramuscular injections of the enzyme hyaluronidase which hydrolyzes hyaluronan, resulted in reduced muscle stiffness and increased the range of passive and active motion in the post-stroke arm with beneficial effects lasting over three months^16,20^.

We used T_1ρ_ mapping to image GAG content in upper limb muscles. T_1ρ_ contrast is an endogenous MRI contrast mechanism that refers to the spin lattice relaxation time constant in the rotating magnetic field, and measures the transverse magnetization decay in the presence of a spin-lock radiofrequency (RF) field^21^. T_1ρ_ contrast is sensitive to lower energy interactions^22^ related to the chemical exchange between extra-cellular water and macromolecules, and is well-suited to characterize proteoglycans^23^. T_1ρ_ mapping allows quantification of GAG content, and has been used to quantify proteoglycan content in cartilage^23,24^, muscle^25–27^ and intervertebral discs^28,29^.

The purpose of this study was to i) provide imaging evidence of increased GAG content in patients with post-stroke muscle stiffness; and ii) to determine the effect of hyaluronidase treatment on intramuscular GAG content.

## Results

**Figure 1:**
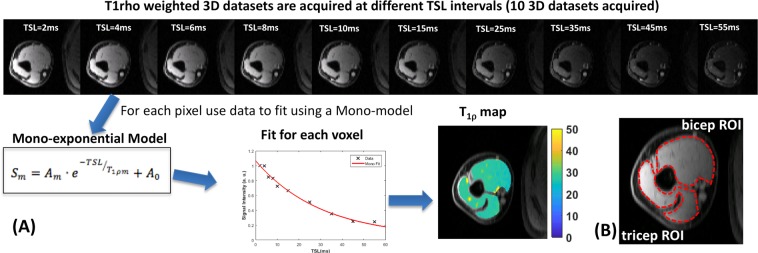
Schematic representation describing the T_1ρ_ mapping process. (**A**) The T_1ρ_ weighted images from all TSL durations are fit on a pixel by pixel basis using a 3 parameter fit mono-exponential model to fit the data in each voxel to produce a T_1ρ_ map. (**B**) Shows typical manually drawn ROIs for biceps and triceps muscles.

Figure 1(A) shows a schematic representation of the data processing pipeline for T_1ρ_ mapping. The data from multiple spin lock times (2–55 ms) are used to generate the T_1ρ_ maps. Data for each pixel across all spin lock times are modeled using a 3-parameter non-linear, mono-exponential decay, and the resulting T_1ρ_ relaxation time for each pixel is displayed as a T_1ρ_ map. Regions of interest (ROIs) for the biceps and triceps muscles are manually drawn (Fig. 1(B)).

### Controls vs patients

**Figure 2:**
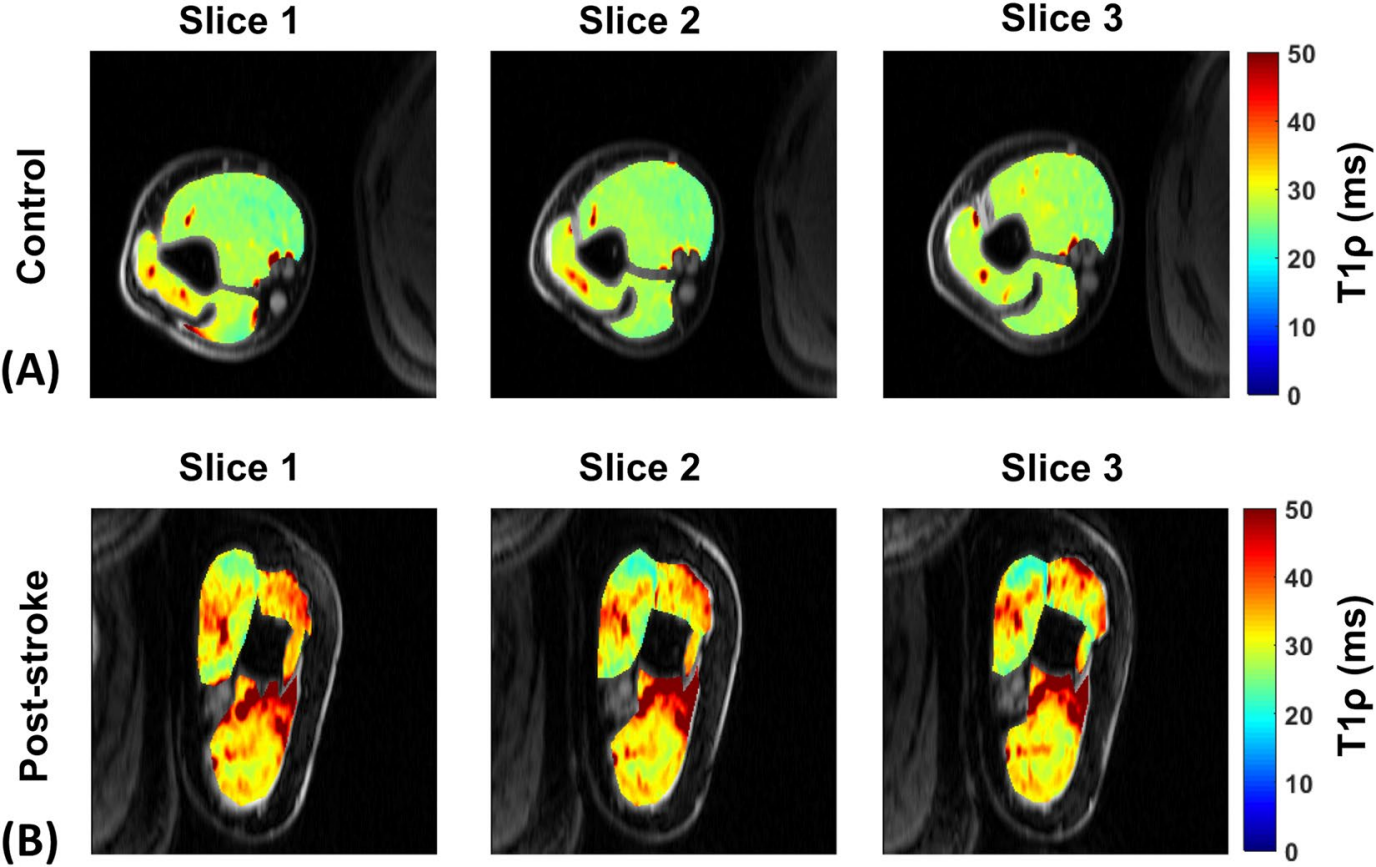
T_1ρ_ maps for a representative control subject and patient (pre-treatment). (**A**) Shows T_1ρ_ maps of 3 representative slices overlaid over anatomy in a control subject. (**B**) Shows T_1ρ_ maps of three representative slices overlaid over anatomy in a patient with post-stroke muscle stiffness prior to hyaluronidase injection treatment.

Figure 2(A) shows representative T_1ρ_ maps for three slices in a control subject. Figure 2(B) shows three representative T_1ρ_ map slices of the affected arm of a patient with post-stroke muscle stiffness before treatment. In the controls, the mean T_1ρ_ relaxation time in the biceps was 26.7 ± 0.54 ms, and in the triceps it was 30.29 ± 2.23 ms. In the patients, prior to treatment, the mean T_1ρ_ relaxation time in the biceps was 34.04 ± 4.39 ms and in the triceps it was 34.06 ± 4.62 ms. In the biceps, a significant difference was observed between the controls and the patients pre-injection (P = 0.006), while the triceps did not show a significant difference (P = 0.14).

### Patients-pre- vs post-treatment

**Figure 3:**
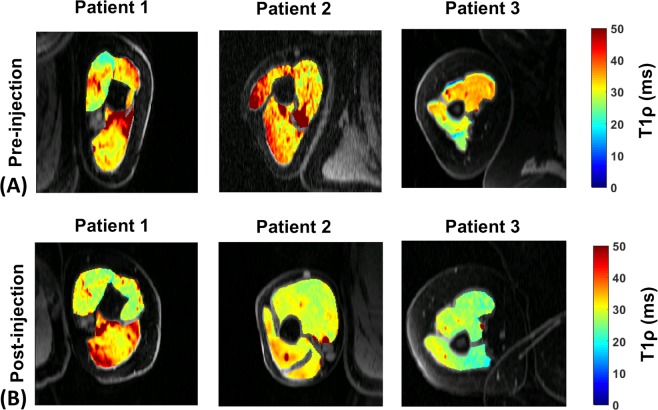
Representative T_1ρ_ maps before and after hyaluronidase injection treatment. (**A**) Show T_1ρ_ maps of representative slices overlaid over anatomy from 3 patients with post-stroke muscle stiffness before injections were administered (**B**) Shows T_1ρ_ maps of the same patients in (**A**) at approximately similar slice locations following hyaluronidase injection treatment. Note the difference in shape of the muscle before and after the injections.

Pre- vs post-treatment analysis used only the patients who came for post-treatment imaging (n = 3). Figure 3 shows representative T_1ρ_ maps in the three patients before injection (A) and after the hyaluronidase injections (B). The slices shown in (A) and (B) correspond to approximately similar slice positions in the affected arm.

The mean T_1ρ_ relaxation time before injection treatment in this cohort for the biceps was 35.48 ± 3.38 ms and for the triceps was 35.36 ± 4.93 ms. Following the hyaluronidase injections, the mean T_1ρ_ relaxation time in the biceps decreased to 29.45 ± 1.23 ms and the mean value in the triceps decreased to 32.91 ± 4.9 ms. A significant difference was observed between pre- and post-injection conditions for the biceps (P = 0.077), but not for the triceps (P = 0.65). To account for the small number of patients in the pre- vs post injection analysis, we reduced the significance level for this comparison to 0.1.

**Figure 4:**
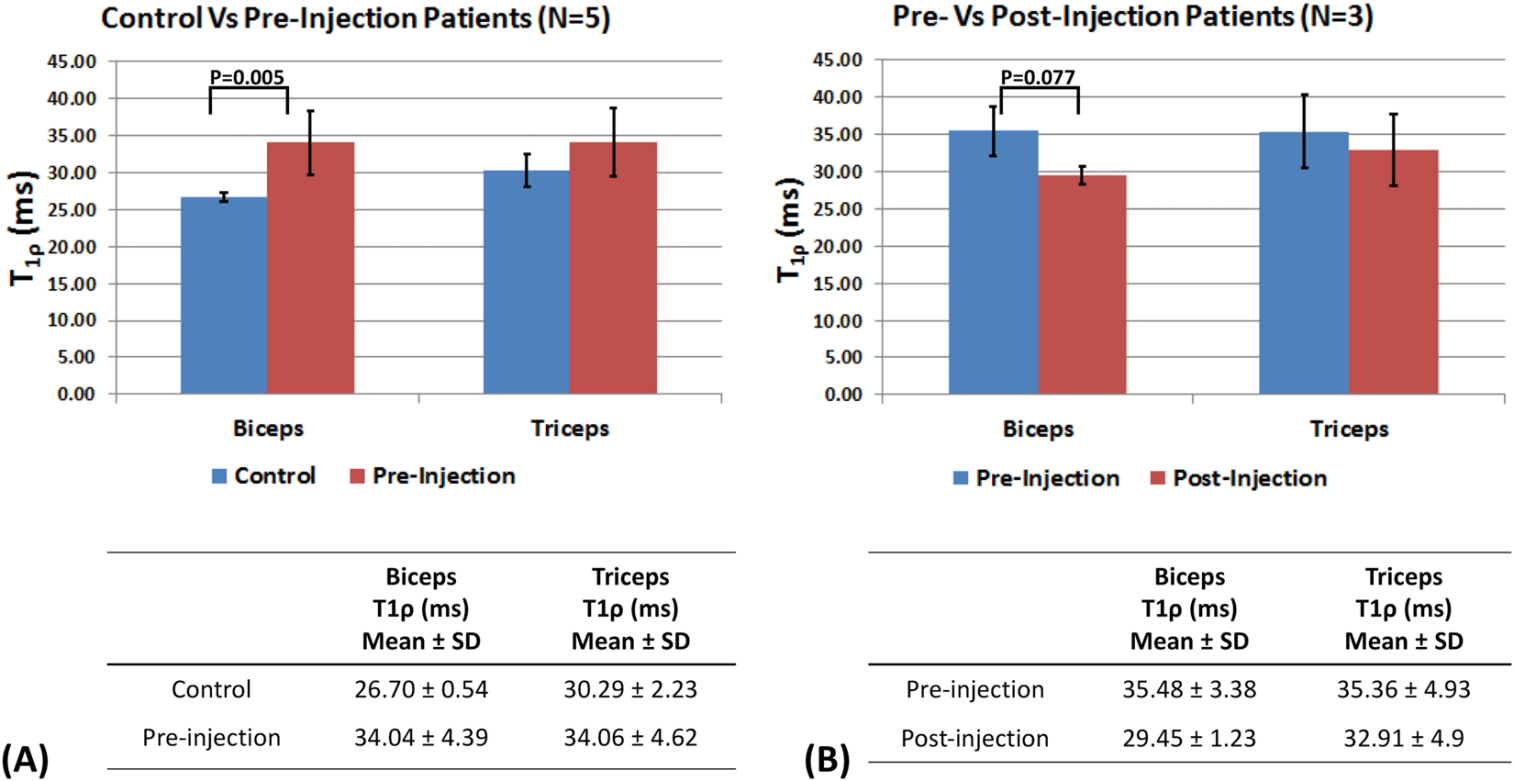
Comparison of mean T_1ρ_ values between groups. (**A**) Shows the comparison of mean T_1ρ_ values in the biceps and triceps ROIs between controls and pre-injection patients (N = 5). The T_1ρ_ values expressed as mean ± SD are shown. In the biceps ROI, there is a significant different in the mean T_1ρ_ value (P = 0.005), while a significant difference in the triceps ROI is not observed (P = 0.14) (**B**) Shows the comparison of mean T_1ρ_ values in the biceps and triceps ROIs between pre- vs post-injection patients expressed as mean ± SD values. For the biceps ROI, a significant difference was observed between pre- and post-injection patients (P = 0.077), but a significant difference was not observed for the triceps ROI between pre- and post-injection (P = 0.65). Significance level set to 0.1, owing to the lower sample size (N = 3).

Figure 4A shows the comparison between controls and pre-injection patients, and Fig. 4B shows the comparison between pre-and post-injection conditions. Table 1 summarizes the results in the biceps and triceps.

**Table 1:** Demographic Information.

Patient Number	Age (years)	Gender	Time since Stroke (months)
1	47	M	96
2	49	M	7
3	47	F	27
4	61	F	45
5	54	F	15

### Dixon water-fat imaging

**Figure 5:**
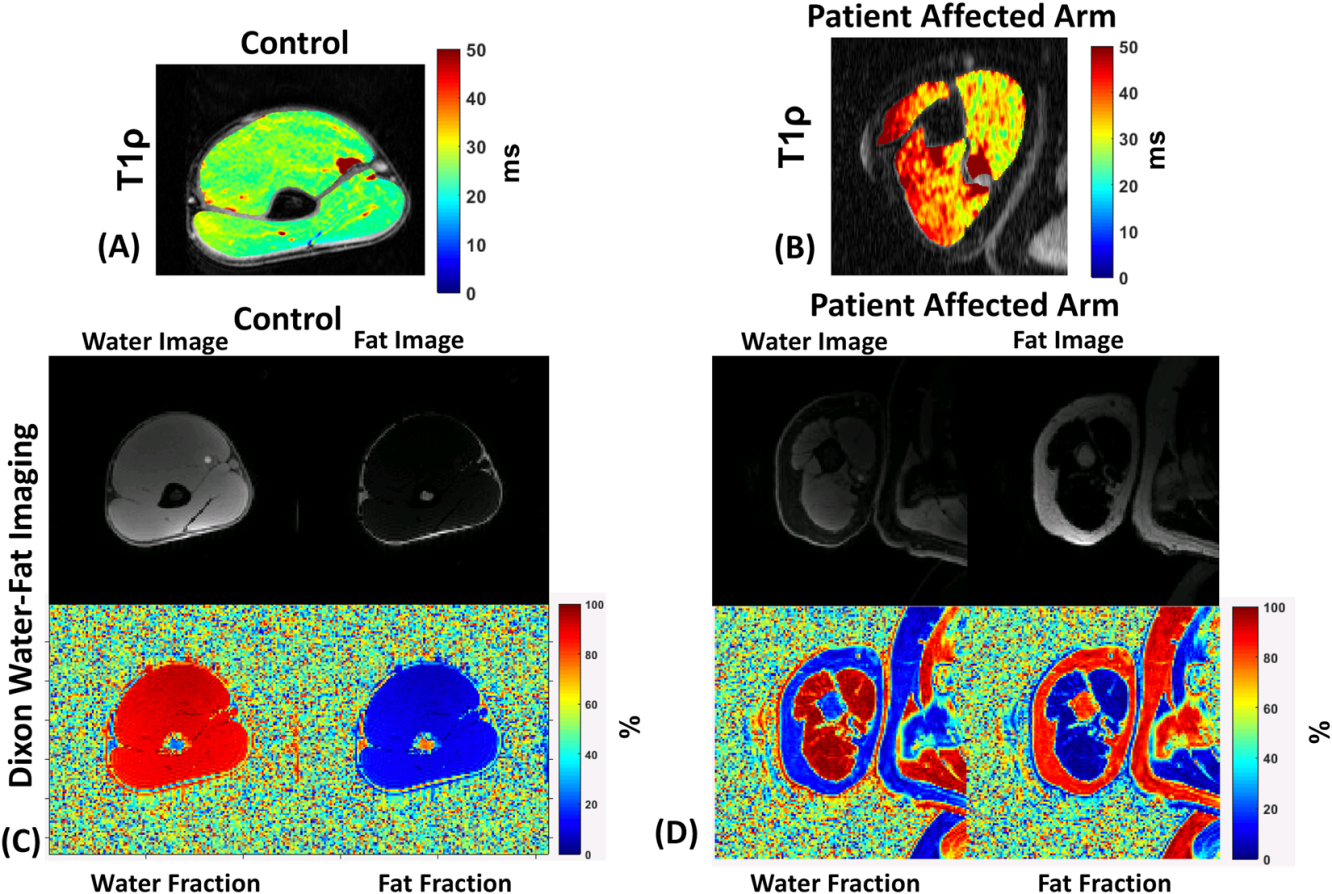
Assessment of fatty infiltration in upper arm muscles using Dixon water-fat imaging. (**A**) Shows the T_1ρ_ map of a healthy control subject, (**B**) shows the T_1ρ_ map of patient before treatment (**C**) shows the calculated results from the Dixon water-fat imaging showing the water image, fat image, water fraction and fat fraction in the control subject in A and in the patient in B before treatment (**D**). Although the triceps and biceps ROIs showed increases in fat fraction in the skeletal muscle of patients, the differences were not significant compared to controls (P = 0.063 for biceps, P = 0.19 for triceps) suggesting that fatty infiltration did not play a big role in the elevation of T_1ρ_ values.

Figure 5 shows the results from the Dixon water-fat imaging. Figure 5(C) shows the Dixon water-fat imaging in a healthy volunteer showing the water image (a), fat image (b), water fraction (c) and fat fraction (d) expressed as a percent. Figure 5(D) shows the same results from the affected arm of a patient prior to treatment. Although both the biceps and triceps showed higher fat fractions in patients, a significant difference between the fat-fractions of the control subjects and patients was not observed in the biceps (P = 0.063), and triceps (P = 0.19) (Table 2).

**Table 2:** Table summarizing data analysis for T1ρ imaging and Dixon water-fat imaging in the control subjects and patients.

	T1ρ (ms) Biceps		T1ρ (ms) Triceps	
	Mean ± STD	95% CI	Mean ± STD	95% CI
**T1ρ Imaging: Controls vs Patients Pre-injection (N** = **5)**				
Controls	26.70 ± 0.54	26.22–27.17	30.29 ± 2.23	28.34–32.24
Patients Pre-Injection	34.04 ± 4.39	30.20–37.89	34.06 ± 4.62	30.01–38.11
P-value: Controls vs Pre-Injection Patients	0.0059		0.1388	
**T1ρ Imaging: Patients Pre- Vs Post-injection (N** = **3)**				
	**Mean ± STD**	**90% CI**	**Mean ± STD**	**90% CI**
Patient Pre-Injection	35.48 ± 3.38	32.26–38.69	35.36 ± 4.93	30.68–40.04
Patient Post-Injection	29.45 ± 1.23	28.28–30.62	32.91 ± 4.9	28.26–37.57
P-value: Pre- Vs Post-injection	0.0767		0.6452	
**Dixon Water-Fat Imaging**				
	**Biceps**		**Triceps**	
	**Controls**	**Patients**	**Controls**	**Patients**
Mean ± STD of % Fat-Fraction	5.07 ± 0.95	7.67 ± 1.77	5.17 ± 1.38	6.52 ± 1.70
95% CI	3.75–6.39	4.41–10.92	3.26–7.08	3.40–9.64
P-Value: Controls Vs Patients	0.063		0.19	

The T_1ρ_ values are shown as mean ± SD and confidence intervals (CI), in the biceps and triceps ROIs. Dixon water-fat imaging results are shown as the mean fat-fraction in controls vs patients expressed as a percentage in the biceps and triceps ROIs. Mean fat fraction percentage, and CI are shown.

### Model of evolution of muscle biochemical and structural changes post-stroke

**Figure 6:**
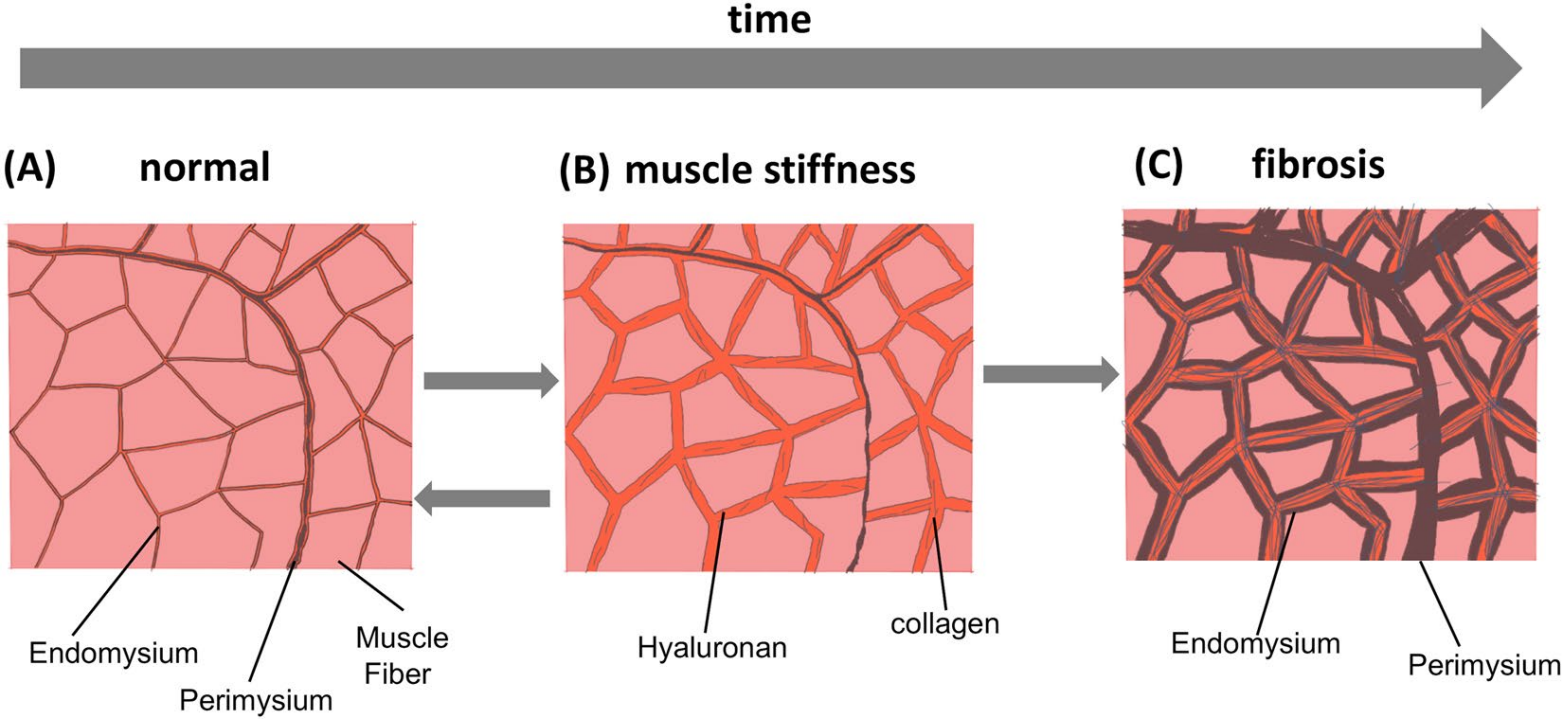
Model of the evolution of muscle biochemical/structural changes post stroke. (**A**) Shows a cross-section of a normal skeletal muscle, (**B**) shows the evolution of muscle stiffness following post-stroke paralysis and immobility that results in accumulation of hyaluronan in the extracellular space, increasing the viscosity of the ECM, and causing the muscle fibers to stick together (**C**) shows that continued inflammation, immobility and muscle stiffness initiates fibrosis leading to the replacement of hyaluronan by collagen, thickened endomysium and perimysium, and muscle fiber atrophy. Note that while (**C**) depicting fibrosis is an irreversible stage, data from literature suggests that the processes between stages depicted in (**A**) and (**B**) are reversible and may represent a potential therapeutic target.

Figure 6 shows a schematic representation of the evolution of the arm muscle micro-environment post-stroke. In normal skeletal muscle, the ECM consists of a thin layer of collagen fibers types I and III, which form the endomysium surrounding each muscle fiber, the perimysium around muscle fiber bundles, and the epimysium surrounding the entire muscle. A thin layer of hyaluronan is present adjacent to the collagenous endomysium, perimysium and epimysium^17^, and its thickness is continuously remodeled in response to mechanical stimuli (Fig. 6A). Following neural injury, inflammation, skeletal muscle paralysis and immobility can lead to an imbalance in the turnover of the ECM resulting in increased hyaluronan production and reduced degradation, and the accumulation of hyaluronan in the ECM; this can increase the viscosity of the ECM causing the muscle fibers to stick to one another which can increase the resistance to movement (Fig. 6B). This is the stage of muscle stiffness. At this stage, the collagen content of the endomysium and perimysium may not be dramatically different from normal and disuse may lead to minor muscle atrophy. Continued inflammation, immobility and/or disuse of muscles due to reduced cortical input and muscle stiffness can initiate a complex pathologic pathway involving transforming growth factor (TGF-β), Rho associated protein kinase (ROCK) and mitogen associated protein kinase (MAPK) resulting in fibrogenesis^30–32^. The excess hyaluronan in the ECM is then replaced by collagen, leading to permanent and irreversible thickening of the endomysium and perimysium, and alteration in the structure and function of the muscle^14^ manifested as contracture (Fig. 6C). This stage may be characterized by marked muscle atrophy.

## Discussion

In this study, we demonstrate that T_1ρ_ MR imaging can be employed to assess the GAG content in muscles post stroke in comparison to control subjects. The study characterized the effect of hyaluronidase injections post stroke, and provides the first *in vivo* imaging evidence for the hyaluronan hypothesis of muscle stiffness.

This study used T_1ρ_ imaging to quantify the amount of GAG in the upper arm muscles. The main advantages of using this T_1ρ_ imaging technique are that it is a completely non-invasive method which does not employ exogenous contrast agents, thus making it safe and well-suited to image patients with stroke, with the added advantage that repeated imaging can be conducted at multiple time points for longitudinal monitoring. Furthermore, T_1ρ_ imaging is sensitive to the chemical exchange of large macromolecules such as hyaluronan with protons in bulk water making it a useful tool to quantify GAG content in muscles before and after treatment. Importantly, it can shed light on the underlying mechanisms of the vexing problem of muscle stiffness after neural injury.

Hyaluronan is a non-sulfated negatively charged polysaccharide composed of repeating chains of two monosaccharides: ß(1,4)-N-acetyl-D-glucosamine and ß(1,3)-D-glucuronic acid, with a molecular formula of C_33_H_54_N_2_O_23_. T_1ρ_ mapping reflects the chemical exchange of protons in water with the hydroxyl (-OH) groups of hyaluronan. *In vivo*, depending on the local environment, T_1ρ_ contrast may depend on both exchangeable (chemical exchange rate, pH, concentration, *f*) and non-exchangeable (dipole-dipole and scalar interactions) mechanisms. In addition, inflammation may also affect T_1ρ_ contrast. Supplementary Fig. 1 shows a schematic diagram of a two-pool model of exchangeable macromolecule proton pools with bulk water affecting T_1ρ_ contrast. Each pool is characterized by its own properties (relaxation rate R_1_, R_2_), solute concentration (*f*_B_) and the chemical exchange between the pools (*k*_BA_, *f*_B_* *k*_BA_).

*In vivo*, increased T_1ρ_ values reflect greater deposits of hyaluronan in the extra-cellular space of the muscles, noted in both the biceps and triceps post-stroke. The increased standard deviation of T_1ρ_ values observed reflects the variability in patients compared to the control subjects. A number of factors may affect these values such as the concentration of hyaluronan, degree of muscle atrophy, presence of fibrosis, and duration of muscle stiffness in individual patients. In Fig. 3, along with reduced T_1ρ_ values, we note the difference in the shape of the muscles among all patients in the pre-injection vs post-injection images. We attribute this to reduced viscosity in the ECM of the muscle after hyaluronidase injections and relaxation of the muscle as a result of reduced hyaluronan content. In Fig. 4, although there were reductions post-treatment in mean T_1ρ_ numbers for both the biceps and triceps, a larger effect was observed in the biceps. Limited elbow extension post-stroke is attributed to stiffness in the biceps, which is typically treated more aggressively^33^. Our results demonstrate that hyaluronan accumulates in both the flexor and extensor compartments, and that both muscle groups may require treatment as visualized using imaging.

Intervention at the stage of muscle stiffness to reduce the imbalance in turnover of the ECM represents a potential therapeutic target. Local intramuscular injection of hyaluronidase into the affected muscles hydrolyzes the excess hyaluronan and reduces the viscoelasticity of the ECM in the affected muscle. Thus muscle stiffness may be reversible with the administration of hyaluronidase, but further studies are needed to investigate its therapeutic potential. The results shown here suggest that using T_1ρ_ imaging as a tool to quantify GAG content and monitor treatment response shows significant potential to enhance the management of post-stroke muscle stiffness, which is a daunting and difficult problem to treat. The results also support the use of hyaluronidase in improving muscle characteristics on T_1ρ_ imaging in patients with post-stroke muscle stiffness.

This study has the following limitations. The patient cohort used in this study is small. Further studies are underway to quantify GAG content in patients with post-stroke muscle stiffness which will include a larger diverse patient population to assess the robustness, repeatability and reproducibility of this technique. The imaging sequence used is not commonly available in all medical centers and may be seen as a barrier to entry for clinical adoption, and may require requesting the vendor for imaging sequences that can perform T_1ρ_ imaging. Nevertheless, this imaging technique for the quantification of GAG can easily be translated to other disorders that result in muscle spasticity and stiffness to understand the non-neural contributions of changes in the ECM, such as in multiple sclerosis, traumatic brain injury, cerebral palsy, and spinal cord injury.

In summary, this study provides T_1ρ_ MR imaging evidence of accumulation of GAGs, specifically hyaluronan, in the upper limb muscles of patients with post-stroke muscle stiffness.

## Methods

### Study design

This study was approved by New York University Langone Health’s institutional review board (IRB) and was health insurance portability and accountability act (HIPAA) compliant. This was a prospective, non-randomized imaging study to determine intramuscular GAG content using proton T_1ρ_ relaxation mapping. All subjects gave written informed consent after explanation of the study and the protocol, as per the IRB guidelines.

### Subjects

Five post-stroke patients with upper limb muscle stiffness were recruited for this study (2 males/3 females, age = 52 ± 5 years). The patients were English speaking, adults, with chronic post-stroke moderate-to-severe muscle stiffness (Table 1). The patients did not have any contraindication to MRI (MR non-compliant implants, pacemakers, or claustrophobia). Five healthy volunteers (1 male/4 females, age = 27 ± 2 years) with no known history of mental illness, or physical injury to the arms, without contraindication to MRI were also recruited.

### Study protocol

For the patients a pre-injection MRI scan (N = 5) of the affected upper arm was performed. Then they received off-label intramuscular hyaluronidase injections (Hylenex, Halozyme Therapeutics, Inc) for clinical treatment of their muscle stiffness unrelated to the imaging research study. The intramuscular dose of hyaluronidase was similar to those reported in a previous article and included several muscles besides the imaged arm muscles^16^. Approximately 1–4 weeks following the injections, the patients were scanned again using the same MRI protocol. For post-injection imaging, only 3 of the 5 injected subjects were scanned. Only the subjects having pre- and post-injection MRI scans were included in the pre- Vs post-treatment analysis. Healthy volunteers were scanned once using the same MRI protocol as the patients.

### Imaging protocol

All imaging scans were done on a clinical 3 T MRI scanner (Prisma, Siemens Healthineers, Erlangen, Germany). An 8-coil flexible receive array coil was wrapped around the arm. A 3D-turbo-FLASH (fast, low angle shot) MRI sequence with a customized T_1ρ_ preparation module was used to enable varying spin lock durations (TSL). A paired self-compensated spin-lock pulse was used to minimize B_0_ and B_1_ variations^34^. The sequence parameters included FOV = 130 mm, matrix size = 256 × 64 × 64, TR = 1500 ms, resolution = 0.5 × 2 × 2 mm^2^, spin-lock frequency = 500 Hz, 10 TSL durations = 2, 4, 6, 8, 10, 15, 25, 35, 45, 55 ms. acquisition duration = ~10 minutes (Fig. 2). The MRI body coil was used for transmission, and vendor supplied flexible receive array coils (8 coil elements each) were wrapped around the affected arm (right arm for controls) for imaging. To investigate if fatty infiltration in skeletal muscle played a role in patient results, Dixon based methods were used to separate fat and water distribution using the iterative decomposition of water and fat with echo asymmetry and least-squares estimation (IDEAL)^35^ technique. The Dixon water/fat imaging parameters included: TR = 9.3 ms, TE = (2.26, 3.08, 3.90) ms, FOV = 180 mm, matrix size = 128 × 128, resolution = 1.4 × 1.4 mm^2^, total acquisition time ~ 4.5 min.

### Data analysis and statistics

Manual ROIs were drawn for the biceps and triceps muscles. The mono-exponential T_1ρ_ mapping was performed by fitting the signal intensity at different spin-lock durations for each pixel using a three-parameter non-linear mono-exponential model:

1 $$S=A\cdot {e}^{-TSL/{T}_{1\rho }}+{A}_{0}$$

where S is the signal intensity, A is the amplitude, TSL is the spin lock duration, T_1ρ_ is the mono-exponential relaxation time in the rotating frame, and A_0_ is the average noise level.

For the Dixon water-fat imaging, IDEAL post-processing was used to separate the water and fat images from the 3-echo images. Water-fractions and fat-fractions were calculated from the water and fat images. The fat-fraction maps were used to quantify the fat infiltration in the muscles.

The mean values and standard deviations in the manually drawn ROIs were calculated across the patients and controls. Wilcoxon Mann-Whitney test was used for comparing the pre- vs post-treatment conditions in patients’ vs controls. A P-value of less than 0.05 was used for analysis as the threshold to reject the null hypothesis. For the comparison between pre and post-injection patients, a P-value of 0.1 was used due to the small number of subjects.

## Supplementary information

Supplementary Material

## Data Availability

The datasets generated during and/or analyzed during the current study are available from the corresponding author on reasonable request.
