# Supplementary material for: Quantifying muscle glycosaminoglycan levels in patients with post-stroke muscle stiffness using T1ρ MRI
Source: Sci Rep. 2019 Oct 10;9:14513. doi: 10.1038/s41598-019-50715-x (PMC6787087; doi:10.1038/s41598-019-50715-x)
Supplement: Supplementary file 1 — Supplementary Material [file 41598_2019_50715_MOESM1_ESM.docx]

**SUPPLEMENTARY MATERIAL**

Quantifying muscle glycosaminoglycan levels in patients with post-stroke muscle stiffness using T_1ρ_ MRI

**Authors:** Rajiv G. Menon, Ph.D., Preeti Raghavan, MD, Ravinder R. Regatte, Ph.D

**
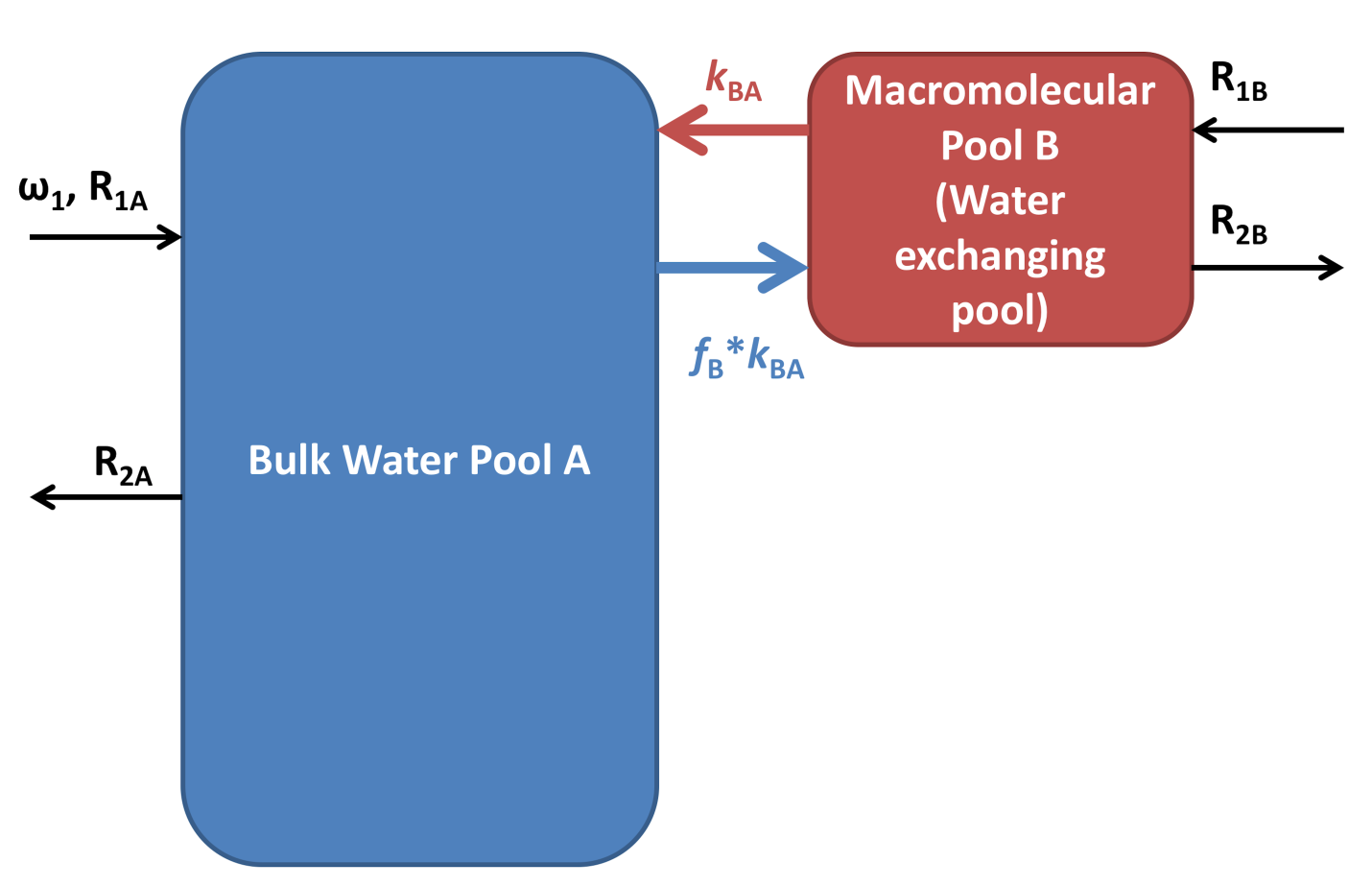
**

**Supplementary Figure 1.** Schematic representation of the chemical exchange between bulk water and macromolecular pools.
